# Brazilian Psychiatric Association Consensus for the Management of Acute Intoxication: general management and specific interventions for drugs of abuse

**DOI:** 10.47626/2237-6089-2022-0571

**Published:** 2024-05-24

**Authors:** Leonardo Baldaçara, Amanda de Gouvêa Pettersen, Verônica da Silveira Leite, Flávia Ismael, Carolina Pereira Motta, Railson Alves Freitas, Nicoli Abrazo Fasanella, Lucas Alves Pereira, Maria Elisa Lima Barros, Leonardo Barbosa, Ana Luiza Silva Teles, Ruy Palhano, Helio Penna Guimaraes, Maria Aparecida Braga, João Mauricio Castaldelli-Maia, Carla Bicca, Analice Gligliotti, Ana Cecilia Petta Roseli Marques, Antônio Geraldo da Silva

**Affiliations:** 1 Universidade Federal do Tocantins Palmas TO Brazil Universidade Federal do Tocantins, Palmas, TO, Brazil.; 2 Associação Brasileira de Psiquiatria Rio de Janeiro RJ Brazil Associação Brasileira de Psiquiatria, Rio de Janeiro, RJ, Brazil.; 3 Universidade de São Caetano do Sul São Caetano do Sul SP Brazil Universidade de São Caetano do Sul, São Caetano do Sul, SP, Brazil.; 4 Centro de Atenção Psicossocial Porto Nacional TO Brazil Centro de Atenção Psicossocial, Porto Nacional, TO, Brazil.; 5 Hospital Regional de Porto Nacional Porto Nacional TO Brazil Hospital Regional de Porto Nacional, Porto Nacional, TO, Brazil.; 6 Instituto Tocantinense Presidente Antonio Carlos Palmas TO Brazil Instituto Tocantinense Presidente Antonio Carlos (ITPAC), Palmas, TO, Brazil.; 7 Centro de Atenção Psicossocial – Álcool e Drogas III Palmas TO Brazil Centro de Atenção Psicossocial – Álcool e Drogas III, Palmas, TO, Brazil.; 8 Pontifícia Universidade Católica de São Paulo São Paulo SP Brazil Pontifícia Universidade Católica de São Paulo, São Paulo, SP, Brazil.; 9 Escola Bahiana de Medicina e Saúde Pública Salvador BA Brazil Escola Bahiana de Medicina e Saúde Pública, Salvador, BA, Brazil.; 10 Universidade Salvador Salvador BA Brazil Universidade Salvador, Salvador, BA, Brazil,; 11 Centro Universitario UniFG Guanambi BA Brasil Centro Universitario UniFG, Guanambi, BA, Brasil.; 12 CAPS II Beija-Flor Guanambi BA Brasil CAPS II Beija-Flor, Guanambi, BA, Brasil.; 13 Centro de Ensino Unificado de Brasília Brasília DF Brazil Centro de Ensino Unificado de Brasília (UniCEUB), Brasília, DF, Brazil.; 14 Associação Brasileira de Medicina de Emergência Porto Alegre RS Brazil Associação Brasileira de Medicina de Emergência, Porto Alegre, RS, Brazil.; 15 Departamento de Neurociências Centro Universitário Faculdade de Medicina do ABC Santo André SP Brazil Departamento de Neurociências, Centro Universitário Faculdade de Medicina do ABC, Santo André, SP, Brazil.; 16 Departamento de Psiquiatria Faculdade de Medicina Universidade de São Paulo São Paulo SP Brazil Departamento de Psiquiatria, Faculdade de Medicina, Universidade de São Paulo, São Paulo, SP, Brazil.

**Keywords:** Poisoning, emergencies, drug abuse, detoxification, acute intoxication

## Abstract

**Objectives:**

To present the Brazilian Psychiatric Association’s Consensus on the Management of Acute Intoxication.

**Methods:**

A group of experts selected by the Brazilian Psychiatric Association searched for articles on the MEDLINE (by PubMed) and Cochrane databases, limited to human studies and acute intoxication. Working groups reviewed these materials for appropriateness to the topic and the quality of the work. A survey was conducted using the Delphi method to produce a table of agreed recommendations presented at the end of the systematic review. Three survey rounds were held to reach consensus.

**Results:**

Support for intoxication should start with Initial Management: Resuscitation/Life Support/Differential Diagnosis. For this, the group proposed the following sequence of assessments: A (airway), B (breathing), C (circulation), D.1 (disability), D.2 (differential diagnosis), D.3 (decontamination), D.4 (drug antidotes), E (enhanced elimination). The group of experts then presented specific interventions for the main drugs of abuse.

**Conclusions:**

Management of intoxication with drugs of abuse is complex and requires systematic protocols. The group suggests adoption of the A-B-C-D-E technique first, with constant investigation. Then, specific conduct and support until remission of intoxication. The literature is still scarce in evidence on the subject. Therefore, this consensus was necessary. We believe that at present this document can help psychiatric, general, and emergency physicians deal with emergency psychiatric episodes due to acute intoxication. This work could stimulate future studies on the topic.

## Introduction

Acute intoxication is a clinically significant transient condition that develops during, or shortly after, consumption of a drug and is characterized by disturbances in consciousness, cognition, perception, affect, behavior, and/or coordination.^1,2^ These changes are caused by known pharmacological effects of substances in the brain and their intensity is closely related to the amount consumed. They are time-limited and abate as the drug is cleared from the body.^1,2^ Presenting features may vary for each substance and can evolve into several complications, from physical damage (toxic hepatitis, seizures, cardiac arrhythmias) to cardiorespiratory arrest, agitation with aggression, car accidents, and suicidal behavior.^1,2^

Poisoning is probably one of the leading causes of admission to emergency departments (EDs) and intensive care units. The frequency of death ranges from 0.05% (United States) to 4% (South Africa).^3^ Data from the World Health Organization (WHO) report mortality rates from unintentional poisoning ranging from 0.001 to 5.45 per 100,000 inhabitants worldwide.^4^ Poisoning is also the preferred method of suicide, suicide attempt, and self-harm.^5^ In England, poisoning accounts for 5-10% of ED workload.^6^

In Brazil, between 2010 and 2014, 376,506 suspected cases of poisoning were registered on the Notifiable Diseases Information System (Sistema de Informação de Agravos de Notificação [SINAN]), although the true magnitude is not yet fully known. Notification of Exogenous Intoxications became mandatory as of 2011, when exogenous intoxication (EI) was added to the list of compulsory notification diseases.^7^ Another study analyzed compulsory notifications for EI in Brazil between 2007 and 2017 using SINAN data. Of 833,282 cases of EI, 54.25% were women and 54.47% were between 15 and 39 years old. The injuries were recorded mainly in the urban area (86.3%) of the Southeast Region (47.65%).^8^

In 2018, an estimated 269 million (range: 166-373 million) people had used a drug at least once in the previous year, equivalent to 5.4% (range: 3.3-7.5%) of the global population aged 15-64.^2^ Assuming no change in the global prevalence of drug use, considering solely the projected increase in the global population would result in the global number of people who use drugs rising by an estimated 11 percent, to 299 million people by 2030. The health consequences of drug use can include a range of negative outcomes such as drug use disorders, mental health disorders, human immunodeficiency virus (HIV) infection, hepatitis-related liver cancer and cirrhosis, overdose, and premature death.^9^

Despite being a frequent situation in emergencies, EI is still a cause of great difficulties for physicians.^10,11^ The literature is extensive, but provides little evidence. Lack of training and stigma related to mental illness are factors that further hinder the care of these patients.^12^ Therefore, documents that standardize care of such cases could help health professionals act more effectively and help with planning of public prevention policies.

This work aims to standardize information on management of patients in acute intoxication from drugs of abuse and present it for physicians, especially psychiatrists.

## Methods

The group’s initial objective was to develop a guideline and score studies according to the 2011 Oxford evidence levels. However, after a detailed search, not enough articles were found with sufficient evidence to accomplish this task. As an alternative, a Consensus document was developed. Procedures focused on discussion and integration of findings from peer-reviewed published research on the topic. Working groups then reviewed these materials for their appropriateness to the topic and study quality. A survey using the Delphi method was then conducted to construct a table of agreed recommendations at the end of the systematic review.

### The panel of experts

A group of experts selected by the Brazilian Psychiatric Association, based on publications or clinical experience in psychiatric emergencies, medical emergencies, or substance abuse disorders.

### Eligibility criteria

Inclusion criteria for the literature research included papers published (or in press) on adults (18 years old), from 2010 to 2020. Editorials, narrative reviews, small naturalistic studies, case reports, animal or in vitro studies, and letters to the editor were excluded. However, other manuscripts that did not meet the inclusion criteria were assessed when needed, including association guidelines, government documents, and articles published outside the search period.

### Search strategy

**Figure 1:**
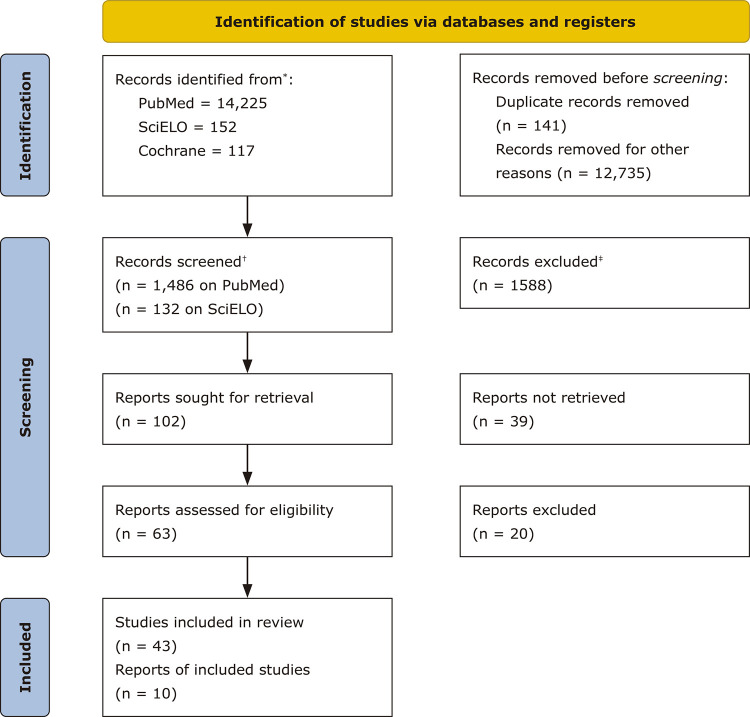
The Preferred Reporting Items for Systematic Reviews and Meta-Analysis (PRISMA) 2020 flow diagram.

Searches for articles were run on the MEDLINE (by PubMed), SciELO, and Cochrane databases, limited to human studies and acute intoxication. Keywords used were poisoning OR emergencies OR drug abuse OR detoxification OR acute intoxication AND management (Figure 1).

### Selection process

First, two panel members, LB and AGP, searched for abstracts and selected the most relevant. Second, LB, AGP, CPM, and RAF analyzed full manuscripts to select the most important and those of the highest quality.

### Data collection

This process was conducted by LB, AGP, CPM, and RAF.

### Data items

LB, AGP, CPM, and RAF wrote an outline of the manuscript. Items included for discussion were selected by another panel of experts in the first phase of the Delphi process.

### Delphi method

We conducted a survey using the Delphi method to construct a table of agreed recommendations at the end of the systematic review.^13^ Three survey rounds were conducted to develop a consensus. The first survey included open-ended questions at the end of each section, inviting participants to add comments and suggestions by e-mail. Later rounds were conducted online. The survey was sent to the members of the acute intoxication task force for anonymous responses. Panel members rated survey items, ranging from “essential” to “should not be included.” We calculated the proportions of respondents rating each item. Survey items were classified as endorsed, re-rated, or rejected. The method used to conduct this survey is the same one used to develop the clinical recommendations presented in the Assessment and management of agitation in psychiatry: expert consensus.^14^

#### Endorsed items

Items rated by the panel of experts as “essential” or “important” were included in the recommendations.

#### Re-rated items

Items rated as “essential” or “important” by 65-79% of panel experts were included in the next survey for re-rating after considering feedback from first-round results. Panel members could decide whether they wanted to maintain or change their earlier rating on these relatively controversial items. Items were re-rated only once; if they still did not achieve the criterion for endorsement, they were rejected.

#### Rejected items

Items that were not included by at least 65% of panelists in the first round were rejected and excluded. The first survey included 102 items. The second survey included 57 items. The briefer third survey consisted of eight items that needed re-rating. Fifty-two were endorsed and formed the final manuscript in the item results.

## Results and discussion

### Initial management: Resuscitation/Life support/Differential diagnosis

All situations of acute intoxication must be taken extremely seriously. Screening and hospitalization will be necessary when intentional self-poisonings or recreation ingestion occur. If symptoms are initially unexplained, urinary drug screening may be indicated, although it is rarely helpful for short-term decisions.^11,15-17^

The patient physical examination verifies the main signs and symptoms related to each intoxication condition, which, when grouped, can characterize a certain toxic syndrome.^11,15^

**Figure 2:**
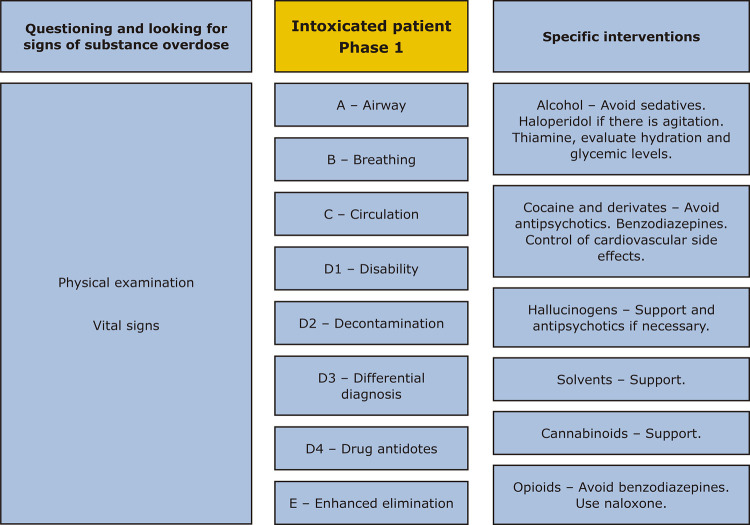
Flow diagram for the management of acute intoxication.

Initial assessment of the patient must go through specific steps,^16,17^ as outlined in Figure 2.

#### A. Airway

The airway must be kept patent through positioning, suction, or insertion of an artificial nasal or oropharyngeal airway. If there are signs of coma or depressed airway reflexes, perform endotracheal intubation or insert an extra-glottic device (supraglottic or retroglottic).^17^

#### B. Breathing

- Clinically assess the quality and depth of respiration and assist, if necessary, with a bag valve-mask device or mechanical ventilator.^17^ Arterial blood PO_2_ analysis may reveal hypoxemia, which may be caused by respiratory depression, bronchospasm, pulmonary aspiration, or noncardiogenic pulmonary edema, while CO_2_ partial pressure is useful in assessing the adequacy of ventilation. When using a pulse oximeter to assess oxygenation, one should be cautious, because some may cause misinterpretations, affected by ambient brightness, shock, and peripheral tissue perfusion.^17^

#### C. Circulation

- Measure the pulse and blood pressure and estimate tissue perfusion (e.g., by measurement of urinary output, lactate, skin signs, arterial blood pH). Use an electrocardiogram (ECG) for continuous monitoring. Collect blood, intravenously, to analyze levels of glucose, electrolytes, serum creatinine, liver enzymes, and possibly perform quantitative toxicology tests.^17^
- Arrhythmia may occur, depending on different substances and reasons, such as hypoxia, metabolic acidosis, electrolyte imbalance (e.g., hyperkalemia, hypokalemia, hypomagnesemia, or hypocalcemia), following exposure to chlorinated solvents or by chloral hydrate overdose. Atypical ventricular tachycardia (torsades de pointes) is often associated with drugs that prolong QT interval or conditions associated with hypomagnesemia.^17^
- Various medications can cause hypotension, such as antihypertensives, beta-blockers, calcium channel blockers, disulfiram (ethanol interaction during treatment), iron, trazodone, quetiapine, and other antipsychotic and antidepressant agents. Poisons that cause hypotension include cyanide, carbon monoxide, hydrogen sulfide, aluminum or zinc phosphide, arsenic, and certain mushrooms. When this symptom occurs, most patients respond to empirical treatment with repeated intravenous boluses of 200 mL or 4 mL/kg of 0.9% saline or other isotonic crystalloids up to a total of 1-2 L in 1 h.^17^
- Some intoxications can cause hypertension. The most common substances in this case are amphetamines and synthetic stimulants, anticholinergics, cocaine, performance-enhancing products (e.g., caffeine, phenylephrine, ephedrine, or yohimbine), monoamine oxidase (MAO) inhibitors, and other drugs. It is important to consider which antihypertensive to use, if necessary, considering the mechanism of action and which adrenergic receptors will be blocked.^17^

#### D.1. Disability

- Seizures are always generalized and usually respond to benzodiazepines with barbiturates as second line. Phenytoin is ineffective.^16^
- Drug-induced syndromes such as malignant hyperthermia, serotonin syndrome, and neuroleptic malignant syndrome should be investigated.^12^
- Glucose levels should be checked and treated if the value is < 4 mmol/L (hypoglycemia link).^16^ Once hypoglycemia is confirmed, in all obtunded, comatose or convulsive patients, give 50% dextrose, 50-100 mL by intravenous bolus.^17^
- Consider thiamine reposition and intravenous fluids if chronic alcohol consumption or signs of malnutrition.^11,17-21^
- If there is hyperthermia, consider poisoning by stimulants, atropine salicylates, strychnine, 2,4-dinitrophenol, antidepressants, antipsychotics, and various other medications.^11,17-21^
- If there is hypothermia and coma, consider poisoning by opioids, ethanol, hypoglycemic agents, phenothiazines, barbiturates, benzodiazepines, and other central nervous system (CNS) agents.^11,17-21^

#### D.2. Differential diagnosis

The “5 Ws” strategy is used in the clinical interview of the patient and/or their companions for initial assessment of an intoxicated patient. The “5 Ws” refer to data related to the patient (Who?) – obtain the patient’s history of diseases, medications in use, previous suicide attempts, occupation, access to substances, drug use, and pregnancy; to the substance used (What?) – find out which substance was used and the quantity; to time of occurrence (When?) – check the time of exposure and for how long the substance was used, in cases of repeated exposures; to place of occurrence (Where?) – where the exposure occurred and if bottles, packages, syringes, or pill packs were found close to the patient, check which medicines the patient has access to; and to the reason for the exposure (Why?) – identify the circumstance of the exposure, since it is extremely important to know whether it was a suicide attempt, homicide, accident, recurrent episodes of drug abuse, or others.^11,15^ Basic support should be provided for all patients as described below. However, ongoing management will depend on the type of substance used, and so it is essential to identify it. Four measures can be used to find out what substances are involved: 1) Questioning the patient and their companions; 2) Finding substances or documents in their clothes; 3) Indirectly, by the signs or symptoms of identification; or 4) through toxicology tests.^11,15^

#### D.3. Decontamination

In case of contact with eyes, irrigate with saline solution.^17^ In case of skin contamination, remove clothes and wash with plenty of soap and water.^22^

Regarding the gastrointestinal tract, a variety of methods may be considered, such as *emesis* (no role in the hospital setting) and administration of ipecac at the site of ingestion or in the ED, but should be avoided.^22^ *Gastric lavage* should not be performed routinely, and in situations where gastric lavage might seem proper, consider treatment with activated charcoal and always observe for supportive care.^23^ *Activated charcoal,* used for prompt adsorption of drugs or toxins in the stomach and intestine, is contraindicated in patients with an altered state of consciousness and in intoxication by ethanol/glycols, alkalis/corrosives, metals including lithium, iron compounds, potassium, fluoride, cyanide, hydrocarbons, and mineral acids such as boric acid.^16,17,24^ *Whole bowel irrigation*, is not routinely indicated, but can be considered for cases of potentially toxic ingestion of sustained-release or enteric substances such as coated drugs, drugs not absorbed by activated charcoal (e.g., lithium, potassium, and iron) and for the removal of illicit drugs in “packers” or “stuffers” from the body.^25^

#### D.4. Drug antidotes, see specific guidelines.

Aiming to help in diagnosis and treatment, the possibility of assaying serum drug levels can also be useful for choosing antidotes, if there are any.^11,17^

#### E. Enhanced elimination

- Forced diuresis: forced diuresis is hazardous; the risk of complications (fluid overload, electrolyte imbalance) usually outweighs its benefits.^17^
- Urinary alkalinization: useful for salicylate toxicity if performed meticulously. This practice enhances poison elimination by administering intravenous sodium bicarbonate to produce urine with a pH ≥ 7.5. It may be considered the first choice for patients with moderate to severe salicylate poisoning (with no criteria for hemodialysis). It may also be effective in eliminating chlorpropamide, 2,4-dichlorophenoxyacetic acid, diflunisal, fluorine, mecoprop, methotrexate, and phenobarbital (activated charcoal can be used as a second choice option for phenobarbital if urinary alkalinization is not used). The most common complication is hypokalemia, but it can be corrected with administration of potassium.^26^
- Urinary acidification^17^: sometimes used for intoxication with amphetamines, and phencyclidine, but it is not remarkably effective and is contraindicated if rhabdomyolysis or myoglobinuria are present.
- Multi-dose activated charcoal: should only be considered in cases with ingestion of potentially fatal amounts of carbamazepine, dapsone, phenobarbital, quinine, or theophylline. Despite well-known pharmacokinetics, there is no evidence of improvement in clinical outcomes.^27^
- Dialysis: for known or suspected potentially lethal amounts of a dialyzable drug, poisoning, deep coma, apnea, or in patients with severe kidney, cardiac, pulmonary, or hepatic disease who will not be able to eliminate toxins by the usual mechanisms.^16,17^ Although the drugs discussed in this article do not present indications for dialysis, the intoxicated individual may use medications for other purposes, which increases the number of complications and damage. Examples of dialyzable substances include barbiturates, lithium, methylxanthines (theophylline and caffeine), metformin, valproic acid, and carbamazepine.
- Attention: whenever there are doubts, contact the Information Centers. If there is insufficient infrastructure and team to provide support, consider transferring to a tertiary center.

## Specific interventions

### Alcohol

Alcohol is a substance commonly used all over the world, mainly in Western countries. It also constitutes the oldest and most widespread substance of abuse.^28^ Ethanol is a biphasic psychoactive substance, a CNS depressant whose effects of intoxication can vary according to the dose, type of drink ingested, speed of ingestion, genetic factors, and consumption pattern (degree of tolerance of the user).^10,11^ The manifestations of intoxication are heterogeneous, affecting neurological, cardiac, gastrointestinal, pulmonary, and metabolic functions. Among the many alcohol-related problems referred to emergency units, acute intoxication is the most common, mainly in adults and adolescents.^10,11^

**Signs and symptoms.** Ethyl breath, conjunctival hyperemia, altered speech, altered motor coordination, impaired attention, altered ability to discern, altered affect (euphoria, joy, sadness, irritation), behavior change, ability to cooperate, and presence of nystagmus^1,28-30^ can be seen.

An alcoholic drink is considered to have 14 g of alcohol. Consumption is abusive when it exceeds four drinks per day or eight drinks per week for women and five drinks per day or 15 drinks per week for men.^1,28-30^ Use is considered compulsive or binge drinking when, in a period of 2 h, there is the consumption of four or more drinks in the case of women and five or more drinks in the case of men. People over 60 years old should avoid drinking more than one dose per day or seven doses per week, with no difference between genders.^1,28-30^

A high dose of alcohol detrimentally affects several functions associated with the prefrontal and temporal lobes, including planning, verbal fluency, memory, complex motor control, and inhibitory mediated motion repulsion.^31,32^

For differential diagnosis, see Table 1.

**Table 1:** Conditions that can mimic acute alcohol intoxication and should be considered as differential diagnoses

Intoxication by other substances/Intoxication by alcohol other than ethanol
Cocaine, opioids, cannabis
Barbiturates
Benzodiazepines
Tricyclic antidepressants
Disulfiram
Carbon monoxide
Metabolic causes
Hepatic encephalopathy
Hypoglycemia
Electrolyte disturbances (hypo/hypernatremia, hypo/hypercalcemia)
Alcoholic ketoacidosis
Diabetic ketoacidosis
Non-ketotic hyperosmolar coma
Uremia
Hypertensive encephalopathy
Infectious diseases
Sepsis
Meningitis
Encephalitis
Neurological causes
Alcohol withdrawal syndrome
Wernicke-Korsakoff syndrome
Cerebrovascular disease
Epilepsy
Trauma
Intracranial bleeding (subdural hematoma)
Concussion
Respiratory causes
Hypoxia
Respiratory depression
Others
Hypotension
Hyper / hypothermia
Hyper / hypothyroidism
Dehydration

**Factors that can influence the severity of alcohol intoxication.** The amount of alcohol ingested, body weight, gender and tolerance to alcohol, alcoholic percentage of the drink, length of time since the drink was ingested, the association of alcohol with opioids and/or benzodiazepines, and presence of food in the stomach. When complementary test for alcohol s are unavailable, the level of intoxication can be assessed according to clinical criteria. Sometimes, there are clinical conditions associated with alcohol poisoning, and laboratory exams and CNS imaging must be considered.^1,10,11,28-30^

**Complications.** Falls and consequent traumas (mainly head traumas), vomiting with bronchoaspiration, cardiac arrhythmias, decompensated cardiac output, hypertensive crises, car accidents, respiratory depression, hypothermia agitation, aggression, triggering mood and psychotic episodes, death.^10,11,28^Table 2 shows the severity of alcohol levels.

**Table 2:** Serum alcohol levels and clinical repercussion^20^

< 50 mg/dL
Deficiency in some tasks that require skills
Increased speech
Relaxation
> 100 mg/dL
Change in feeling of the environment
Ataxia
Hyperreflexia
Impaired judgment
Lack of coordination
Change in mood and behavior
Nystagmus
Extended reaction time
Slurred speech
> 200 mg/dL
Amnesia
Diplopia
Dysarthria
Hypothermia
Nausea and vomiting
> 400 mg/dL
Respiratory depression
Coma
Death

Lethal dose is variable; it can occur in individuals “without tolerance” with serum levels of 300 mg/dL.^20^

**Management of alcohol intoxication.** The treatment of acute ethanol intoxication begins with immediate interruption of alcohol consumption and protection of the airways in all cases, considering that the main clinical complication is respiratory depression. Aspiration prevention is mandatory and positioning the patient laterally can be useful.^28^ Hydration and intravenous glucose should only be administered if the patient has dehydration or hypoglycemia.^10,11^ Administration of thiamine is recommended for all patients with alcohol-related disorders, considering that it is difficult to detect hidden thiamine deficiency and Wernicke’s encephalopathy, which constitutes an increased risk.^28,33^

In cases of mild to moderate intoxication, checking of vital signs is indicated and if there is evidence of dehydration, intravenous fluid administration. The patient must be kept under observation, whether in hospital or an outpatient setting, to detect signs of alcohol withdrawal. Psychomotor agitation and aggressiveness are common in patients with severe intoxication and, in these situations, use of intramuscular injectable haloperidol in monotherapy is indicated, but after verbal de-escalation.^15,28,34^ It is important to avoid benzodiazepines and antihistamines,^23^ with the risk of interaction effects with alcohol. In extreme cases of agitation, when the patient is a risk to himself or others, mechanical restraint may be necessary. Antiemetic medications can be useful in case of nausea and/or vomiting.^28^ Effects of acute intoxication are associated with a greater possibility of situations of violence, in addition to the considerable risk of infection by sexually transmitted diseases.^21,28,29,35-37^

In case of severe intoxication, with a semi-comatose or comatose state, mechanical ventilatory support is necessary, treating hypoglycemia (with 5% glucose solution) and correcting hydro electrolytic disorders, if present, and administration of vitamins, including thiamine.^21,28,29,35-37^

### Cocaine and other stimulants

Cocaine, benzoylmethylecgonine is an alkaloid extracted from the leaves of *Erythroxylon coca*.^38,39^ Cocaine increases the activity of monoamine neurotransmitters in the central and peripheral nervous system by blocking reuptake pumps (transporters) of dopamine, norepinephrine, and serotonin.^38,39^

In addition to the dangers of cocaine overdose and the effects of acute intoxication, use of cocaine is associated with a greater likelihood of situations of violence, and to an elevated risk of infection by sexually transmitted diseases.

**Signs and symptoms.** The usual symptoms of cocaine intoxication include a general excitatory state, feeling of euphoria, increased energy, agitation, aggression, anxiety, restlessness, hallucinations, and delusions. On physical examination, it is possible to observe mydriasis, diaphoresis, tachycardia, hyperthermia, hypertension, tremors, or convulsions. Paranoid delusions, associated with anxiety and psychomotor agitation, are also common. Special attention should be paid to clinical symptoms, especially chest pain, dyspnea, decreased level of consciousness, seizures and hyperthermia, due to the risk to the patient’s life.^11,17,40^

**Complications.** The main cardiovascular complications of acute cocaine poisoning are acute myocardial infarction, cardiac arrhythmias, coronary aortic dissection, aortic rupture, and myocarditis. Neurological complications are seizures, hemorrhage, intracranial infarction, altered mental status, and spinal cord infarction.^10,11,39,40^ Other common complications we should be alert for include severe hypertension, acute renal failure, hyperthermia, pneumothorax, and deep vein thrombosis.^10,11,39,40^

**Management of intoxication by cocaine and other stimulants.** The treatment for cocaine intoxication must consist of vital and symptomatic support according to the patient’s symptoms. Antidotes are not yet known. Screening tests for psychoactive substances are also rarely available in emergency units in Brazil. In addition to careful physical examination, if possible, a detailed history taking is recommended and it is also important to obtain more information about the condition from family members, friends, the removal team who brought the patient to the hospital, or the police authority who accompanied the patient. The first action is to diagnose and treat any eventual organic changes that could put the patient’s life at risk. Special attention should be given to the cardiovascular and neurological systems due to specific fatal impairments.

In case of mild cocaine intoxication, in which the patient does not have any warning signs such as chest pain, severe hypertension, major increase in heart rate, or signs of neurological impairment, one should only observe progress, keeping the patient in a safe place, with regular evaluation, and without environmental stimuli. In patients with severe intoxication, the initial priority involves clinical support of the patient, mainly protecting the airways and maintaining oxygenation and ventilation, in addition to obtaining venous access.^38,40^ For patients with moderate psychomotor agitation who are collaborative, have no warning signs indicating immediate risk, and have no significant changes to vital signs, benzodiazepines are recommended, such as diazepam orally at a dose of 5-10 mg, which can be repeated according to the patient’s progress. In patients with intense psychomotor agitation and/or aggressiveness, the recommendation is to use benzodiazepine intravenously (diazepam) or intramuscularly (midazolam). The option most available in Brazil is diazepam 0.2-0.3 mg/kg/dose, by slow infusion, without dilution. In cases lacking intravenous access, the choice is midazolam 0.2-0.7 mg/kg administered intramuscularly. In some cases of severe agitation, to protect the patient, mechanical restraint may be necessary for the shortest time.^38,40^

Haloperidol can also be used in cases of intense psychomotor agitation and paranoid delusions, but special attention should be paid to its cardiovascular effects, the decrease in the seizure threshold, and a possible increase in heart temperature. This medication should be avoided in people who have seizures, hyperthermia, severe hypertension, or cardiac arrhythmias; in these cases, use of benzodiazepines should be prioritized.^11,15,17^ Phenothiazine antipsychotics such as chlorpromazine should be avoided due to the significant reduction in the seizure threshold and the potential to trigger cardiac arrhythmias. Use of beta-blockers should be avoided in patients who have used cocaine in the past 24 h, due to its potential to trigger a reduction in blood flow and increase severe coronary events.^10,11,34^

The challenge for the physician in the ED is to identify patients at risk who would benefit from a specific intervention. All patients with acute coronary syndrome, especially young men, without risk factors other than smoking, should be asked about cocaine usage.^38^ First-line treatment of a patient with cocaine-related chest pain compatible with myocardial ischemia and ST-segment elevation consists of administration of oxygen and sublingual nitroglycerin or verapamil. If there is no response, immediate coronary angiography should be performed. Both nitroglycerin and verapamil have been shown to reverse cocaine-induced hypertension, coronary arterial vasoconstriction, and tachycardia.^38^ Beta-blockers (especially non-selective β-blockers) are relatively contraindicated in cocaine-associated acute coronary syndrome. Beta-receptor blockade causes unopposed α-receptor stimulation, which may lead to aggravation of coronary arterial vasoconstriction and systemic hypertension. Some authors recommend labetalol, a joint α- and β-blocker. However, labetalol is a non-selective β-blocker with only modest α-blocking properties.^38^ Thrombolysis should only be given when a thrombus has been shown on angiography or if pharmacological treatment has failed and angiography is not possible. Administration of naloxone and flumazenil should be avoided since they may lead to severe complications.^38^

In severe and resistant cases, there may be a need for patient intubation for better clinical stabilization and protection of the airway, in which case benzodiazepines or propofol should be prioritized. Use of succinylcholine in intubation is contraindicated in these cases.

### Hallucinogens

This group includes serotonin 2A receptor (5-HT2AR) agonists (lysergic acid diethylamide, psilocybin, and N,N-dimethyltryptamine); mixed serotonin and dopamine reuptake inhibitors and releasers (3,4-methylenedioxy-methamphetamine); N-methyl-D-aspartate antagonists (ketamine and dextromethorphan); kappa opioid receptor agonist salvinorin A; and anticholinergics.^41,42^

**Signs and symptoms.** Drug-induced conditions associated with perceptual changes are generally accompanied by physiological abnormalities. With hallucinogens, sympathomimetic effects are common, occur shortly after ingestion, and usually precede the hallucinogenic effects.^42,43^ Delirium or psychosis, however, is also observed with other drugs that show similar effects, such as phencyclidine (PCP), amphetamines, cocaine, and anticholinergics.^42^ Patients with amphetamine intoxication typically present with elaborate and paranoid delusions, as well as visual disturbances. Agitation or extreme agitation along with marked hyperthermia should suggest possible exposure to drugs such as cocaine or PCP.

**Complications.** Induced psychosis, arrhythmias, hyperthermia, agitation and aggressiveness, delirium, coma.^42^

**Management of hallucinogen intoxication.** Support as described previously. The patient should be placed in a quiet room under close observation. Most patients typically need only supportive care.^42^ Support airway and administer oxygen, if comatose. Administer naloxone if concurrent opiate use is suspected. If agitated, combative, or hyperactive, administer benzodiazepines.^34,42^ If benzodiazepines are ineffective, may give haloperidol.^34,42^ Cool, if hyperthermic, and watch for rhabdomyolysis. Substance abuse/detoxification referral and counseling.^42^

## Solvents

The “organic solvents” belong to a group of volatile compounds or mixtures that are relatively chemically stable and exist in a liquid state at 0 °C to 250 °C (32 °F to 482 °F). The most common are classified as aliphatic hydrocarbons, cyclic hydrocarbons, aromatic hydrocarbons, halogenated hydrocarbons, ketones, amines, esters, alcohols, aldehydes, and ethers. There are also mixtures or combinations of chemical compounds. The underlying pathology, biological processes, or modes of action include the release of catecholamines (acute effects of solvents, effects on ion channels [including GABA receptors] in the brain [inhalation anesthetics], and effects on GABA receptors [sedatives]).^44^

**Signs and symptoms.** The acute, transient toxic effects of organic solvent exposure in humans result from the pharmacologic action of the solvent within the CNS.^34^ These effects include CNS depression, psychomotor impairment, narcosis, respiratory failure, drowsiness, headache, dizziness, dyspepsia, nausea, impaired psychomotor functions, impairment of choice reaction time, perceptual and sensory-motor speed, and impairment of body balance. A progression of signs and symptoms includes possible first agitation, progressing to confusion, slurred speech, ataxia, and loss of consciousness.^44^

**Complications.** The main ones are coma, seizures, respiratory arrest, and cardiac dysrhythmias. Cardiac arrest may be the first sign of a high dose of solvent.^44^

**Management.** General life support measures, removal from exposure, and use of antidotes are recommended. Flumazenil can also be used but is not recommended if other toxic agents may be involved.^44^

### Cannabinoids

Cannabis (*Cannabis sativa*) has over 500 natural compounds, including cannabinoids, terpenoids, flavonoids, and alkaloids. Among these, Δ9-tetrahydrocannabinol (THC), the primary psychoactive ingredient, has provoked widespread recreational use and misuse of the plant. Over time, other major plant cannabinoids (termed phytocannabinoids), such as cannabidiol (CBD), cannabichromene, cannabigerol (CBG), cannabidivarin, D9-tetrahydrocannabivarin (THCV), and many others, have also been elucidated.^45^ Today, over 100 phytocannabinoids have been discovered, with THC and CBD being the two most studied cannabinoids.^45^ These phytocannabinoids can interact with the brain’s endogenous cannabinoid system (ECS) to elicit a range of neurobehavioral outcomes, including perturbation to the normal structure and function of the brain. The ECS consists of two main cannabinoid receptors (CB1 and CB2 receptors), endogenous cannabinoids (termed endocannabinoids, with two of the most well-studied being anandamide [AEA] and 2-AG [2-arachidonoylglycerol]) that act as natural ligands at the cannabinoid receptors, and enzymes that participate in their synthesis, uptake, or metabolism (e.g., fatty acid amide hydrolase [FAAH] and monoacylglycerollipase [MAGL]).^45,46^ CB1 receptors are predominantly widely distributed in the CNS, but are also found across peripheral neurons in the cardiovascular, reproductive, and gastrointestinal systems.^45-47^ Within the CNS, their relative densities vary across brain regions. Early autoradiography studies in rodents and humans showed a high concentration of CB1 receptors in the cerebellum, hippocampus, and basal ganglia.^45,47^ They are also densely expressed across the cortex, particularly in the frontal, cingulate, and temporal cortices.^45^ Meanwhile, CB2 receptors are predominantly found in immune cells across the body.^45,46^ While cannabinoids (both endogenous and exogenous) have distinct chemical structures, they all interact with different affinities with the ECS. The widespread distribution of cannabinoid receptors reflects their diverse functions within the brain and body. The endocannabinoids themselves are important signaling molecules that take part in a variety of functions including motor behavior, appetite, emotion, cognition, memory, and sensory, autonomic, and immune functions.^45-47^ Despite some contentious discussions about the addictiveness of cannabis, the evidence shows that long-term use can lead to addiction. Indeed, approximately 9% of those who experiment with marijuana will become addicted.^48^

**Signs, symptoms, and complications.** Regular marijuana use is associated with an increased risk of anxiety and depression,^23^ but causality has not been established. Marijuana is also linked with psychoses (including those associated with schizophrenia), especially among people with a pre-existing genetic vulnerability,^24^ and it also worsens the course of illness in patients with schizophrenia.^48^ Cannabis use impairs critical cognitive functions, both during acute intoxication and for days after use.^48^ Both immediate exposure and long-term exposure to cannabis impair driving ability; cannabis is one of the most frequent illicit drugs reported in connection with impaired driving and accidents, including fatal accidents.^48^ During acute intoxication, the main symptoms that may occur are euphoria, decreased attention, impaired judgment, perceptual changes, changes in sociability, increased appetite, anxiety, impaired short-term memory, and sluggishness. Physical signs include conjunctival hyperemia and tachycardia.^1^ In more severe cases, panic attacks, paranoia, and psychosis can occur.^49^ Psychotic symptoms may occur, but are not necessary for a diagnosis of intoxication, however, insight must be preserved, and psychosis must not be severe or persistent. If psychotic symptoms do persist, the possibility of diagnosing cannabis-induced psychotic disorder should be assessed.^50^

**Management of cannabis intoxication.** The main aim of pharmacological treatment is to improve acute symptoms of intoxication. Thus, when there are intense anxiety symptoms associated with somatic symptoms and panic episodes, benzodiazepines can be used for symptom relief, and use of lorazepam 1 mg every 4 hours is generally sufficient.^51^ In a review article, Gorelick^49^ found a study that reports the subjective improvement of acute effects, tachycardia, and conjunctival hyperemia with use of propranolol up to 120 mg/day, and two clinical trials that show that haloperidol, olanzapine, and risperidone are equally effective in treating the psychosis induced by cannabis intoxication.

No drug currently has a specific action for treatment of intoxication with action on cannabinoid receptors. Studies are still limited, but drugs approved for other uses, such as those mentioned above, can help improve symptoms.^49^

### Opioids

Opioids are substances that increase the activity of one or more G protein-coupled transmembrane molecules, known as mu, delta, and kappa opioid receptors.^52^ These receptors are activated by endogenous peptides and exogenous ligands (morphine) and are distributed throughout the human body. Those that mediate nociception are in the anterior and ventrolateral thalamus, amygdala, and dorsal root ganglia.^52^ Modulation of respiratory responses to hypercarbia and hypoxemia occurs in the brainstem with contributions from dopaminergic neurons, and control of pupillary constriction occurs by receptors in the Edinger-Westphal nucleus of the oculomotor nerve.^52^

**Signs and symptoms.** Changes in mood (predominance of euphoria). The classic syndrome of opioid toxicity includes apnea, stupor, and miosis. However, not all these findings are always present.^52^ The essential symptom of opioid intoxication is respiratory depression. In non-tolerant individuals, therapeutic doses of opioids cause a noticeable decline in all phases of respiratory activity, which is progressive depending on the dose.^52^ In cases of overdose, the decline in respiratory rate is the most noticeable and can progress to apnea.^52^

**Complications.** Coma, respiratory depression,^52^ pulmonary edema, lung injury, hypothermia, seizures, rhabdomyolysis, myoglobinuric renal failure, and compartment syndrome (caused by immobility).^52^

**Management of opioid intoxication.** First, provide support as mentioned previously (A- B- C-D-E), especially respiratory support.^52^ The opioid overdose antidote, naloxone, is a competitive mu opioid-receptor antagonist that reverses all indicators of intoxication. Adults should receive a starting dose of 0.1 to 2 mg of naloxone; if no response occurs, the dose should be raised every 2 minutes according to the schedule, up to a maximum of 15 mg.^43,44^ Orotracheal intubation is an alternative to naloxone administration. Activated charcoal for gastrointestinal decontamination should be reserved for individuals who present within 1 h after ingestion.^43^ Examine the axillae, perineum, scrotum, and oropharynx; any transdermal patches found should be removed, and the skin should be cleaned with soap and cool water.^52^

### Multiple drug intoxication

Management of acute multidrug intoxication is a challenge. First, all conduct should focus on support (A-B-C-D). As for specific procedures, they must only be performed in case of serious effects (of any of the substances) that could put the patient’s life at risk (e.g., severe tachyarrhythmia or severe hypertensive crisis due to severe cocaine intoxication associated with acute alcohol intoxication). In the case cited as an example, use of benzodiazepines, vasodilators, and antiarrhythmics should be considered and monitoring should be redoubled.

## Limitations

The Delphi method enabled production of a document based on the opinions of a group of experts. It was not possible to quantify the quality of evidence using the Oxford method, GRADE, or the Amstar instrument. However, the process allowed us to provide standards for decision-making on a topic on which there is little evidence in the literature and allowed us to bring together diverse types of knowledge in a document. The Delphi technique was used to minimize the chances of error and bias.

## Conclusion

The approach to acute poisoning is complex and requires systematic protocols. We suggest adoption of the A-B-C-D-E technique first, with constant investigation. Then, specific conduct and support until remission of intoxication. The literature is still scarce in evidence on the subject. Therefore, this consensus was necessary. We believe that at present this document can help psychiatric, general, and emergency physicians deal with emergency psychiatric episodes due to acute intoxication. This work could stimulate future studies on the topic.
